# Multimorbidity and Quality of Preventive Care in Swiss University Primary Care Cohorts

**DOI:** 10.1371/journal.pone.0096142

**Published:** 2014-04-23

**Authors:** Sven Streit, Bruno R. da Costa, Douglas C. Bauer, Tinh-Hai Collet, Stefan Weiler, Lukas Zimmerli, Peter Frey, Jacques Cornuz, Jean-Michel Gaspoz, Edouard Battegay, Eve Kerr, Drahomir Aujesky, Nicolas Rodondi

**Affiliations:** 1 Insitute of General Practice BIHAM, Faculty of Medicine, University of Bern, Bern, Switzerland; 2 Department of General Internal Medicine, Bern University Hospital, Bern, Switzerland; 3 Institute of Social and Preventive Medicine, University of Bern, Bern, Switzerland; 4 Clinical Trials Unit, Department of Clinical Research, Bern University Hospital, Bern, Switzerland; 5 Division of General Internal Medicine, Department of Medicine, University of California San Francisco, San Francisco, California, United States of America; 6 Department of Ambulatory Care and Community Medicine, University of Lausanne, Lausanne, Switzerland; 7 Service of Endocrinology, Diabetes, and Metabolism, Lausanne University Hospital, Lausanne, Switzerland; 8 Division of Internal Medicine, University Hospital of Zurich, Zurich, Switzerland; 9 Department of Community Medicine and Primary Care, University Hospitals of Geneva, and Faculty of Medicine, Geneva, Switzerland; 10 Veterans Affairs Center for Clinical Management Research, HSR&D Center of Excellence; Department of Internal Medicine, University of Michigan, Ann Arbor, Michigan, United States of America; University of Tolima, Colombia

## Abstract

**Background:**

Caring for patients with multimorbidity is common for generalists, although such patients are often excluded from clinical trials, and thus such trials lack of generalizability. Data on the association between multimorbidity and preventive care are limited. We aimed to assess whether comorbidity number, severity and type were associated with preventive care among patients receiving care in Swiss University primary care settings.

**Methods:**

We examined a retrospective cohort composed of a random sample of 1,002 patients aged 50–80 years attending four Swiss university primary care settings. Multimorbidity was defined according to the literature and the Charlson index. We assessed the quality of preventive care and cardiovascular preventive care with RAND’s Quality Assessment Tool indicators. Aggregate scores of quality of provided care were calculated by taking into account the number of eligible patients for each indicator.

**Results:**

Participants (mean age 63.5 years, 44% women) had a mean of 2.6 (SD 1.9) comorbidities and 67.5% had 2 or more comorbidities. The mean Charlson index was 1.8 (SD 1.9). Overall, participants received 69% of recommended preventive care and 84% of cardiovascular preventive care. Quality of care was not associated with higher numbers of comorbidities, both for preventive care and for cardiovascular preventive care. Results were similar in analyses using the Charlson index and after adjusting for age, gender, occupation, center and number of visits. Some patients may receive less preventive care including those with dementia (47%) and those with schizophrenia (35%).

**Conclusions:**

In Swiss university primary care settings, two thirds of patients had 2 or more comorbidities. The receipt of preventive and cardiovascular preventive care was not affected by comorbidity count or severity, although patients with certain comorbidities may receive lower levels of preventive care.

## Introduction

Although caring for patients with multiple chronic conditions is common for general internists [1], [2] and an increasing burden for healthcare systems [3], most clinical guidelines continue to use a single-disease framework [4]. Physicians may find it difficult to apply recommendations from clinical guidelines [5], when most of them base their conclusions on clinical trials that exclude patients with multimorbidity [6]. While significant resources are spent on clinical trials, much of this acquired knowledge cannot be translated to broader populations suffering from multiple diseases, which may cause preventable harm due to omitted therapies, suboptimal patient outcomes or inefficient use of resources [5]. In addition, research on multimorbidity in primary care is limited because of the lack of a reliable definition of multimorbidity, explaining why its reported prevalence varies widely between 10 and 81% depending on the scores used and populations studied [7], [8].

Concerns about the potential impact of multimorbidity on quality of care has been noted in the past [9], [10] e.g. in the context of diabetes care [9] or psychiatric disorders [11], [12]. The increasing number of diseases with aging [2], [3], the adverse effects of polypharmacy [13], the time constraints of medical visits [2] and the effects of comorbidities on patients’ ability to manage their self-care [9] all seem to reduce the likelihood of high quality care among those with multimorbidity. A higher number of comorbidities was indeed associated with lower provided quality of care in six US primary care practices [14], but in the largest study of almost 8000 US patients, Higashi *et al.* found that quality of care increased with the number of comorbid diseases in three different cohorts of patients (the CQI study, the ACOVE study and a similarly conducted study among US Veterans) [15]. This positive effect persisted after adjustment for confounders and the number of visits. To further examine this controversial issue, we assessed the association between multimorbidity and the quality of preventive care (e.g. weight and blood pressure measurement, alcohol consumption, and smoking cessation counseling, and cancer screening (see Table S1) and cardiovascular preventive care (e.g. diabetes, hypertension, dyslipidemia, see complete list in Table S1). Instead of analyzing the overall quality of care [15], we analyzed specific indicators of preventive care and cardiovascular preventive care, as multimorbidity may affect predominantly these outcomes and may deflect attention away from preventive care more than care for chronic conditions [2]. The second aim was to assess whether psychiatric disorders interfered with the provided care, as suggested by others [11], [12]. We hypothesized that multimorbidity would be associated with reduced quality of preventive care and cardiovascular preventive care, especially in patients with psychiatric disorders.

## Methods

### Study Population

As previously described [16], we abstracted medical records from a random sample of 1002 patients followed by general internists in four Swiss university primary care settings (Basel, Geneva, Lausanne and Zürich) in a retrospective cohort study over 2 years. The sample was randomly selected from electronic administrative data of all patients aged 50 to 80 years followed in 2005–2006. Most patients were cared for by residents in general internal medicine supervised by university attendings (senior physicians), while about 10% of the patients were followed by attendings alone. The selection was limited to this age group to ensure sufficient prevalence of examined indicators (e.g. eligibility for cancer screening). Briefly, among the 1889 patients identified from electronic administrative data, 54 charts could not be found, most likely because the patients left the clinical setting for another practice. 591 had <1 year follow-up in the primary care clinic during the review period, 125 patients had no outpatient visit to a primary care physician (PCPs) during the review period (emergency visits or nurse appointments only) and 117 were followed only in a specialized clinic. We excluded patients who were not followed for at least 1 year to have adequate time and information to assess provided preventive care, as previously described [16]. The final sample included 1002 abstracted medical charts.

This study was approved by the Ethics Committee of Basel, the Human Research Ethics Committee of Geneva, the Human Research Ethics Committee of Vaud, and the Ethics Committee of Zürich, at the sites of Basel, Geneva, Lausanne, and Zürich, respectively. Because of the retrospective cohort design, the approving Institutional Review Boards waived the requirement of patient consent. These data are not publicly available due to protection of data privacy and rights of each institution on their specific quality measurements, but data could be obtained from the corresponding author on request.

### Definition of Multimorbidity

After reviewing the literature, we found no consistent definition or approach to guide the selection of comorbidities [17]. Others have assessed comorbidity lists ranging from 7 different conditions [18] to 46 [19]. We therefore derived a new list of comorbidities based on the largest study by Higashi *et al.* [15] and the Charlson index (Table S2) [20]. The Charlson index estimates the relative risk of death from an increase of one point of the Charlson index and is approximately equal to that from an additional 10 years of age. A score <3 indicates a low additional risk, whereas>6 is a high and >8 very high risk. Additionally, we included psychiatric conditions (e.g. schizophrenia) as an important comorbidity [21] based on a consensus of the above mentioned references and between the authors. The final list contains 17 important comorbidities for ambulatory medicine (Table S3).

### Definition of Quality of Preventive Care

As previously described [16], we examined 37 quality indicators (Table S1) from the RAND's Quality Assessment Tools related to preventive care and to cardiovascular preventive care [22], [23]. The selection of indicators from the RAND's Quality Assessment Tools has been described in our previous article [16]: 14 indicators for preventive care (e.g. physical examination, alcohol and smoking cessation, cancer screening) and 19 for cardiovascular preventive care (hypertension, dyslipidemia, and diabetes mellitus) and for chronic care on coronary artery disease, as it is the most common cause of death in Switzerland [24]. We excluded quality indicators that were not applicable to our settings (e.g. advice to use seat-belt is rarely performed by generalists in Switzerland), indicators that involved information usually not routinely collected in charts in Switzerland or adults aged 50–80 years, or indicators for conditions of low prevalence in our sample (e.g. asthma). For quality indicators involving a repeated assessment (e.g. HbA1c measurement every 3 months), we checked through all consultations in the review period of 2 years to assess whether the quality indicator was met.

### Statistical Analysis

As done in other studies, for each selected indicator of preventive care and cardiovascular preventive care, we calculated the percentage of provided recommended care by dividing the total number of episodes in which recommended care was delivered by the total number of times patients were eligible for indicators (overall percentage method) [25]. If care was refused by eligible patients, it was considered as provided care so that physician-initiated care was taken into account. To summarize the selected indicators, we calculated aggregate proportions of quality of care among the different categories of prevention (physical examination, counseling, screening and immunization) and an overall aggregate proportion for preventive care. The same method of calculation was used to obtain the aggregate proportions of chronic care for hypertension, dyslipidemia and diabetes, and an overall aggregate proportion for chronic care for cardiovascular risk factors, summarizing care for cardiovascular prevention.

We used logistic mixed-effects models to derive proportions of provided care with 95% confidence intervals, crude and adjusted for age, sex, civil status, legal status, occupation, and treatment center. The mixed-effects model was used to account for the multiple assessments within patients, and for clustering of patients within the different treatment centers. We stratified analyses of overall aggregate proportions according to number of comorbidities and Charlson index score, and we subsequently conducted sensitivity analyses for subgroups of high-risk patients according to the following predefined clinical conditions: cardiovascular disease, chronic pulmonary disease, psychiatric disorders (especially depression and schizophrenia), dementia and cancer by comparing each group with the reference group of patients having no comorbidity. We then calculated aggregate proportions, for all eligible patients, and stratified according to number of comorbidities (0–1 comorbidities vs ≥2 comorbidities). Statistical analyses were performed with STATA release 12.1 (Stata Corp, College Station, TX). All p-values were 2 sided.

## Results

### Characteristics of the Patients

Patients had a mean age of 63.5 years, 44% were women and 51% married (Table 1). 75% had hypertension, 62% dyslipidemia, 29% diabetes, while 18% were former smokers and 23% current smokers. Patients had a median number of 10 outpatient visits over 2 years. Regarding comorbidities, 36% had cardiovascular diseases, 29% psychiatric disorders including depression, 26% chronic pulmonary diseases and 14% cancer. Patients with 2 or more chronic conditions were statistically significantly older, more likely to be female, had more outpatient visits, more medications, and more cardiovascular risk factors.

**Table 1:** Patient characteristics: Random sample of 1002 adults aged 50–80 years in four Swiss university primary care settings.

Characteristics	Overall (n = 1002)	0–1 chronic conditions (n = 326)	≥2 chronic conditions (n = 676)	p-value
Age, mean (SD)	63.5 (8.3)	61.7 (8.1)	64.4 (8.2)	<0.001
Range, minimum - maximum	50–80	50–80	50–80	
Women, no. (%)	445 (44.4)	166 (37.3)	279 (62.7)	0.004
Civil status, no. (% per column)				0.69
Single	151 (15.2)	54 (16.8)	97 (14.4)	
Married	506 (51.0)	158 (49.2)	348 (51.8)	
Divorced, separated	233 (23.5)	78 (24.3)	155 (23.1)	
Widow/−er	103 (10.4)	31 (9.7)	72 (10.7)	
Legal Status, no. (% per column)				
Swiss	560 (55.9)	179 (57.8)	381 (58.1)	<0.001
Resident permit	325 (32.4)	89 (28.7)	236 (36.0)	<0.001
Forced migrants	81 (8.1)	42 (13.6)	39 (6.0)	0.741
Occupation, no. (% per column)				<0.001
Employed	285 (29.0)	138 (43.3)	147 (22.2)	
At home or in education	115 (11.7)	31 (9.7)	84 (12.7)	
Unemployed	101 (10.3)	32 (10.0)	69 (10.4)	
Social aid	109 (11.1)	23 (7.2)	86 (13.0)	
Retired	372 (37.9)	95 (29.8)	277 (41.8)	
Number of outpatient visits over 2 years				
Median (interquartile range)	10 (7–15)	8 (6–12)	12 (8–16)	<0.001
Range, minimum-maximum	2–63	2–41	3–63	
Number of medications, mean (SD)	3.9 (2.7)	2.3 (1.9)	4.7 (2.7)	<0.001
Cardiovascular risk factors, no. (%)				
Hypertension	753 (75.2)	158 (21.0)	595 (79.0)	<0.001
Dyslipidemia	622 (62.1)	159 (25.6)	463 (74.4)	<0.001
Diabetes	292 (29.1)	8 (2.7)	284 (97.3)	<0.001
Family history of early CHD^a^	99 (9.9)	33 (33.3)	66 (66.7)	0.864
Smoking status at baseline, no(%)^b^				
Former smokers	177 (17.7)	38 (21.5)	139 (78.5)	<0.001
Current smokers	230 (23.0)	59 (25.7)	171 (74.4)	0.022
Specific subgroups^c^, no. (%)				
Cardiovascular diseases^d^	364 (36.3)	38 (10.4)	326 (89.6)	<0.001
Psychiatric disorders^e^	294 (29.3)	59 (20.1)	235 (79.9)	<0.001
Chronic pulmonary diseases^f^	261 (26.1)	49 (18.8)	212 (81.2)	<0.001
Cancer^g^	142 (14.2)	7 (4.9)	135 (67.5)	<0.001

a Defined as a coronary heart disease (CHD) event in male first-degree relatives <55 years or in female first-degree relatives <65 years.

b A former smoker had stopped smoking ≥6 months before baseline and a current smoker was smoking at baseline or had stopped<6 months before baseline.

c If the patient has a record of ever having the listed condition or risk factor.

d History of transient ischemic attack, cerebral vascular accident, coronary artery disease, angina, myocardial infarction, congestive heart failure or peripheral vascular disease.

e Depression, bipolar disorder, psychosis, schizophrenia, pervasive development disorder.

f Chronic obstructive pulmonary disease (COPD), asthma, sleep apnea syndrome, sarcoidosis, pulmonary hypertension, bronchiectases, interstitial pulmonary disease or global respiratory insufficiency.

g Solid metastatic, solid non-metastatic cancer, lymphoma, leukemia.

### Multimorbidity and Quality of Preventive Care

Only 7.6% of patients had none of the 17 selected comorbidities (Table 2), with a mean of 2.6 (SD 1.9) comorbidities per patient. However, very few patients (2.3%) had 8 or more comorbidities. The mean Charlson index (Table S2) [20] was 1.8 (SD 1.9),: 31.1% of patients had a index of 0, while only 1.4% had a index of >8.

**Table 2:** Number of comorbidities, Charlson index and quality of preventive care and cardiovascular preventive care, analyzed also for specific subgroups.

	preventive care			cardiovascular preventive care		
	eligible patients, no. (%)	adjusted^a^ % (CI)	p-value	eligible patients, no. (%)	adjusted^a^ % (CI)	p-value
**Comorbidities** b						
0	76 (7.6)	77.4 (65.2–86.2)	0.27^c^	19 (2.3)	80.9 (51.9–94.4)	0.11^c^
1	250 (25.0)	75.6 (63.4–84.7)		179 (21.4)	88.7 (76.3–95.0)	
2	245 (24.5)	75.3 (63.1–84.5)		217 (26.0)	87.8 (74.9–94.6)	
3	178 (17.8)	76.7 (64.6–85.5)		171 (20.5)	88.0 (75.1–94.7)	
4	112 (11.2)	78.4 (66.6–86.8)		110 (13.2)	89.7 (78.1–95.5)	
5	49 (4.9)	75.0 (61.2–85.0)		48 (5.8)	85.6 (70.0–93.8)	
6	39 (3.9)	76.2 (62.9–85.9)		38 (4.5)	90.1 (78.4–95.8)	
7	30 (3.0)	81.9 (70.0–89.7)		30 (3.6)	88.1 (74.0–95.1)	
≥8	23 (2.3)	74.8 (60.0–85.4)		23 (2.8)	93.8 (84.4–97.7)	
**Charlson index**						
0	312 (31.1)	76.9 (65.1–85.6)	0.98^c^	239 (28.6)	90.1 (78.8–95.7)	0.25^c^
1	240 (24.0)	76.0 (63.9–84.9)		178 (21.3)	87.7 (74.7–94.5)	
2	159 (15.9)	76.6 (64.5–85.5)		144 (17.3)	86.6 (72.7–94.1)	
3	129 (12.9)	77.6 (65.6–86.3)		124 (14.9)	89.4 (77.6–95.3)	
4	76 (7.6)	77.7 (65.7–86.5)		73 (8.7)	89.3 (77.5–95.3)	
5	34 (3.4)	74.9 (61.2–85.0)		34 (4.1)	88.3 (81.7–95.0)	
6	26 (2.6)	79.1 (66.1–88.0)		23 (2.8)	92.3 (81.7–97.0)	
7	12 (1.2)	80.2 (64.9–89.9)		7 (0.8)	89.4 (72.1–96.5)	
≥8	14 (1.4)	70.1 (54.0–82.5)		13 (1.6)	91.0 (78.0–96.6)	
**Specific subgroups** d						
Cardiovascular diseases^e^	364 (36.3)	78.8 (61.2–89.8)	0.86^f^	364 (36.3)	94.1 (81.3–98.3)	0.23^f^
Chronic pulmonary diseases^g^	261 (26.1)	78.2 (57.0–90.7)	0.84^f^	261 (26.1)	91.8 (68.9–98.3)	0.20^f^
Psychiatric disorders^h^	294 (29.3)	80.9 (60.6–92.1)	0.21^f^	294 (29.3)	79.7 (45.0–95.0)	0.64^f^
Depression	197 (19.7)	76.4 (49.3–91.5)	0.79^f^	197 (19.7)	73.2 (26.8–95.3)	0.32^f^
Schizophrenia^i^	19 (1.9)	35.1 (5.7–82.9)	0.44^f^	19 (1.9)	75.8 (0.0–100.0)	0.52^f^
Dementia	24 (2.4)	46.8 (8.7–89.0)	0.73^f^	24 (2.4)	17.3 (0.0–99.2)	0.97^f^
Cancer^j^^,^ k	142 (14.2)	76.1 (46.3–92.1)	0.77^f^	142 (14.2)	96.7 (74.2–99.7)	0.32^f^

a Data adjusted for these patients characteristics: age, sex, civil status, legal status, occupation and center. In a 2nd model we adjusted also for the number of outpatient visits by performing a Sensitivity analyses, which showed similar results.

b Based on previous studies[16] and the Charlson index [20].

c p for trend.

d If the patient has a record of ever having the listed condition or risk factor.

e History of transient ischemic attack, cerebral vascular accident, coronary artery disease, angina, myocardial infarction, congestive heart failure or peripheral vascular disease.

f p-value comparing adjusted data for each subgroup to patients with 0 comorbidities.

g Chronic obstructive pulmonary disease (COPD), asthma, sleep apnea syndrome, sarcoidosis, pulmonary hypertension, bronchiectases, interstitial pulmonary disease or global respiratory insufficiency.

h Depression, bipolar disorder, psychosis, schizophrenia, pervasive development disorder.

i Not further adjusted for legal status because of low number of patients with data on legal status (n = 10 of 19 patients with schizophrenia).

j Solid metastatic, solid non-metastatic cancer, lymphoma, leukemia.

k Lower care when metastatic cancer only (data not shown, due to small number of 16 patients).

**Figure 1:**
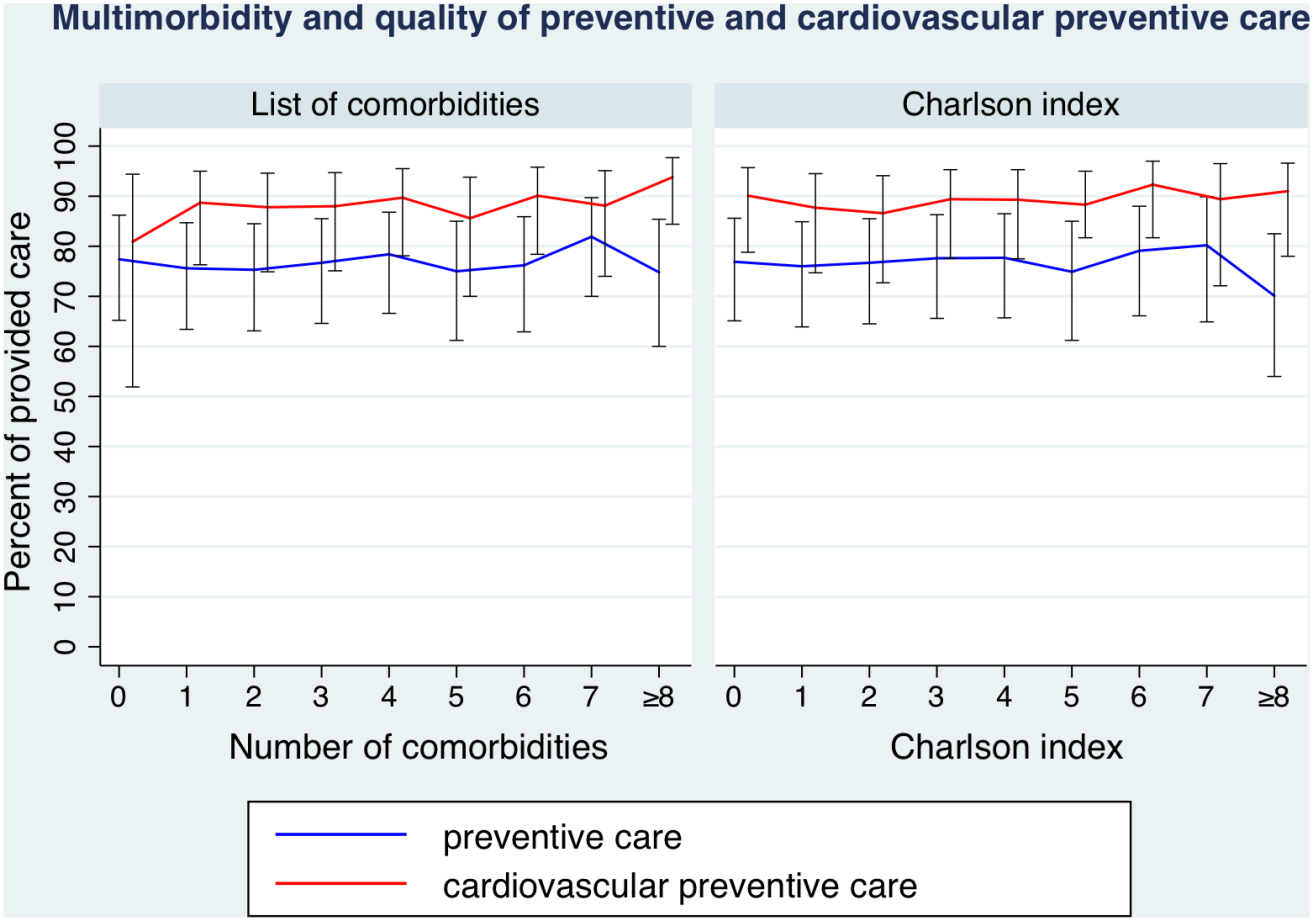
Measures of multimorbidity and association with quality of preventive care. Left part: Number of comorbidities and percent of provided care for preventive care (blue line) and cardiovascular preventive care (red line), bars showing 95% confidence intervals. Right part: Respective analysis with the Charlson index. Data adjusted for age, sex, civil status, legal status, occupation and treatment center. In a second model we adjusted for number outpatient visits and found similar results.

In unadjusted analyses, the global aggregate score for preventive care was 69.2% (95% CI 60.2–76.9) and 83.9% (CI 79.3.87.7) for cardiovascular preventive care (data not shown). The quality of preventive care was not associated with higher numbers of comorbidities or points of the Charlson index (Table 2). Results were similar after adjusting for age, sex, civil status, legal status, and occupation (Figure 1). In a sensitivity analysis, we further adjusted for the number of outpatient visits in the previous 2 years by performing and found similar results (data not shown).

### Analysis of Specific Subgroups of Comorbidities

While quality of preventive care and cardiovascular preventive care were comparable in patients with cardiovascular conditions, pulmonary diseases or cancer, patients with schizophrenia or dementia had a pattern of lower preventive care and patients with dementia had also lower cardiovascular preventive care in adjusted analysis (Table 2). However, the differences were not statistically significant, likely due to the small number or patients (schizophrenia 19, dementia 24). Rates of preventive care were not lower for other psychiatric disorders, including depression.

### Specific Quality Indicators

Patients with 2 or more chronic conditions received statistically significantly more smoking cessation counseling (Table 3). In a sensitivity analysis excluding patients with COPD, asthma or cardiovascular disease, the aggregate score did not decrease with multimorbidity (data not shown).

**Table 3:** Adjusted aggregate scores of provided as recommended preventive care and cardiovascular preventive carea: adjusted data^a^.

Aggregate scores, % (CI)	Overall^b^, % (CI)	0–1 comorbidities, % (CI)	≥2 comorbidities, % (CI)	p-value
Physical examination	97.9 (92.3–99.5)	97.8 (91.7–99.4)	98.3 (93.3–99.7)	0.07
Alcohol consumption counseling	90.6(43.3–99.2)	90.2(42.2–99.1)	91.4 (45.2–99.3)	0.56
Smoking cessation counseling	72.8 (35.5–92.9)	69.6 (32.5–91.6)	78.1 (42.5–94.5)	0.006
Cancer screening	61.4 (26.4–87.6)	62.5 (27.2–88.1)	59.5 (24.6–86.8)	0.39
**Global aggregate score for preventive care**	**76.4 (64.6–85.2)**	**76.2 (64.5–85.1)**	**76.7 (64.9–85.4)**	**0.67**
Diabetes mellitus	80.7 (53.2–93.1)	78.0 (46.8–93.5)	81.5 (54.2–94.3)	0.52
Hypertension	91.3 (75.9–97.2)	90.6 (74.0–97.0)	92.0 (77.3–97.5)	0.20
Dyslipidemia	95.2 (7.1–100)	92.2 (3.6–100)	96.8 (8.7–100)	0.06
**Global aggregate score for cardiovascular preventive** **care** c	**88.6 (76.3–94.9)**	**88.6 (76.2–95.0)**	**88.6 (76.2–94.9)**	**0.97**

a Data were adjusted for age, sex, civil status, legal status, occupation and treatment center. In a second model we adjusted also for the number of outpatient visits by performing a sensitivity analyses and found similar results. Detailed numbers for each indicator are provided in the Table 2 by *Collet et al* [16].

b If care was refused by eligible patients, it was counted as provided care to measure physician-initiated care. When care was provided less frequently than specified (i.e., once a year instead of twice a year or only once instead of annually), it was counted as unprovided care to measure physician adherence to recommendations.

c When care was contraindicated, the patient was not counted as eligible, thus reducing the denominator.

## Discussion

Among a random sample of 1002 patients treated in Swiss university primary care settings, we found very few patients without one or more chronic condition, with rates comparable to patients of similar age in other studies in primary care [26], [27]. We found high rates of preventive care (69%) and cardiovascular preventive care (83%). The quality of preventive care and cardiovascular preventive care was not associated with increasing multimorbidity, either using the number of comorbidities or the Charlson index. Patients with dementia received less preventive care (47%), but so did those with schizophrenia (35%), although the differences were not statistically significant.

Our study showed a comparable stable quality of preventive care despite multimorbidity, as found by Higashi *et al.* [15] in the US setting. However, several differences have to be mentioned. First, the age distribution differed because the previous study included many adults younger than 50 years. Second, Higashi *et al.* used indicators of overall quality, while we focused on preventive care and cardiovascular preventive care specific indicators, assuming that multimorbidity might affect prevention first. Similarly, Heflin *et al.* [28] also found no association between multimorbidity and the receipt of cancer screening among over 2000 patients replying to a US community-based survey. Bae *et al.* [29] demonstrated that diabetic patients with more chronic conditions received better quality of preventive care among 1700 US diabetic patients and suggested this finding was accounted for by the higher rates of outpatient visits. In our present study, results were not confounded by the number of outpatient visits.

Some patient subgroups received less preventive care, such as those with dementia or schizophrenia, although these differences did not reach statistical significance. These results are consistent with a study [11] among 113,000 US veterans showing that patients with psychiatric disorders received less preventive care, especially for immunization and cancer screening. Other studies also reported the lack of cancer screening in mentally ill patients [30]–[32]. In our settings, patients with dementia received less preventive care, which might be appropriate among patients with severe dementia who have a limited life expectancy [33]. However, in principle patients with schizophrenia should receive the same quality of prevention as other adults. It can be speculated that this effect might be due to a competitive issue to address all health aspects during a time-constrained consultation. Thus, schizophrenia could be a so dominant condition that it eclipses other health problems, as previously described by Piette *et al.* [9]. This is particularly of concern for cardiovascular prevention, as many of these patients are at increased risk for metabolic syndrome due to treatment with antipsychotic medications [31]. Additional efforts are needed to deliver adequate preventive care to patients with psychiatric disorders.

How can we explain the consistent high quality of preventive care despite multimorbidity? Higashi *et al.* [15] proposed some potential explanations, such as an increased use of health care in patients with multimorbidity and the fact that adjusting data for this increased use decreased the observed finding of higher quality in patients with multimorbidity. However, in our study the adjustment for the number of outpatient visits did not affect the quality of prevention. The high quality of care could also result from the lack of time to estimate each individual's eligibility for screening, to know and apply all the published guidelines, the lack of specific guidelines for patients with multimorbidity, or the perceived need to provide better care for older patients with multimorbidity [34].

Patients benefit from a universal healthcare coverage in Switzerland. Every Swiss resident is covered by a mandatory health insurance that covers universal healthcare, including adults with low income who receive social aid to cover healthcare costs, regardless of their age or whether they work. Patients are free to choose their primary care physician (PCP). However, high quality of care might not be fully explained by health insurance status only; we have recently found that both forced migrants and undocumented migrants in Switzerland had lower quality of preventive care, albeit forced migrants have health care coverage and undocumented migrants do not [35].

This universal healthcare coverage includes most of preventive care services. Therefore, PCPs do not have to choose between taking care of comorbidities or prevention for cost reasons. However, these high rates of prevention were achieved in Switzerland despite the lack of systematic performance monitoring and annual report cards on quality of care, such as US Healthcare Effectiveness Data and Information Set (HEDIS) [36], [37] or of financial incentives to improve quality.

Our study has several limitations. As previously described [16], our data were only abstracted from medical charts with potential underreporting; it has been found that measurements of quality of care may be about 5% lower using abstraction of medical charts compared to use of clinical vignettes and 10% lower compared to use of standardized patients [38]. Additionally, we could not assess other parameters of socio-economic status, such as income and education, because of the lack of reliable information on these variables in the medical charts. Our data were only abstracted in university primary care settings, where almost all patients received their care from residents at their end of postgraduate training. Therefore, our data may not be generalizable to community-based PCPs. We found only very few studies directly comparing performance between community-based PCPs and university-based physicians in Switzerland. One study among Swiss community-based PCPs found similar results for diabetes care [39]. However, we did not find other studies directly comparing overall performance between community-based PCPs and university-based physicians. Lastly, the prevalence of some comorbidities may be higher in our population than in community-based PCPs, such as for hypertension (75%). However, multimorbidity is also an increasing burden for community-based PCPs [40], [41].

## Conclusions

In summary, multimorbidity is very common in Swiss university primary care settings, as well as for community-based practice[41]. Quality of preventive care and cardiovascular preventive care remains high regardless of increasing number of comorbidities. There was a pattern of appropriately lower prevention among patients with dementia. This was also found in schizophrenic patients, a population that should receive the same preventive care as other healthy adults. Additional efforts should be done to implement adequate preventive care for patients with psychiatric disorders.

## Supporting Information

Table S1 **Selected quality indicators for preventive and cardiovascular preventive care.**
(DOCX)

Table S2 **Weighted index of the Charlson index.**
(DOCX)

Table S3 **List of 17 selected comorbidities in ambulatory medicine.**
(DOCX)
