# Supplementary material for: Multimorbidity and Quality of Preventive Care in Swiss University Primary Care Cohorts
Source: PLoS One. 2014 Apr 23;9(4):e96142. doi: 10.1371/journal.pone.0096142 (PMC3997570; doi:10.1371/journal.pone.0096142)
Supplement: Table S1 — Selected quality indicators for preventive and cardiovascular preventive care. (DOCX) [file pone.0096142.s001.docx]

**Table S1.** Selected quality indicators for preventive and cardiovascular preventive care

| **Quality indicators for preventive care** | **Quality indicators for preventive cardiovascular care** |
| --- | --- |
| **Physical examination** | **Diabetes** |
| Annual blood pressure measurement | Diabetes documented for patients <75 years |
| Weight measurement | Glycosylated hemoglobin (HbA1c) twice a yeard |
| Height measurement | Annual eye and visual exam* |
| **Alcohol consumption counseling** | Cholesterol tests documented |
| Asked about drinking problem | Annual proteinuria |
| Advise to decrease drinking for at-risk or binge drinkers^a^ | Foot examination twice a yeard |
| **Smoking cessation counseling** | Blood pressure documented |
| Smoking status documented | Follow-up visits twice a year |
| Annual advice to quit smoking | Glucose monitoring for diabetics taking insulin |
| Counseling offered to smokers attempting to quit | Dietary and exercise counseling for newly diagnosed diabetics |
| Pharmacotherapy offered to smokers attempting to quit if > 10 cigarettes/day | Oral hypoglycemics for type 2 diabetics who have failed dietary therapy (HbA1c ≥7% after 6 months) |
| Abstinence documented 4 weeks after smoking cessation counseling | Insulin offered to type 2 diabetics who have failed oral hypoglycemics (HbA1c ≥7% with two oral drugs after 6 months) |
| **Cancer screening** | Angiotensin-converting enzyme inhibitor of angiotensin receptor blocker^e^ offered within 3 months after noting proteinuria or microalbuminuria^f^ |
| Screening for colon cancer (age 50-80)^b^ | **Hypertension** |
| Screening for breast cancer (age 50-70)^b^ | Diagnosis of hypertension when 3 separate visits with blood pressure ≥140/90mmHg |
| **Influenza immunization** | Lifestyle modification for hypertensiond |
| Annual influenza vaccine for patients ≥65 years | Annual visit for hypertensive patients |
| Annual influenza vaccine for immunocompromised patients <65 years^c^ | Pharmacotherapy or lifestyle modification for uncontrolled hypertension (≥140/90mmHg over 6 months)d |
|  | **Dyslipidemia** |
|  | Two cholesterol tests before start of therapy |
|  | Cholesterol tests if heart disease and no pharmacological therapy |
|  | **Chronic care for cardiovascular diseases**^g^ |
|  | Aspirin for coronary artery disease |
|  | Beta-blockers after acute myocardial infarction |
|  | Antiplatelet therapy after stroke or transient ischemic attack |
|  | Angiotensin-converting enzyme inhibitor or angiotensin-receptor blocker^e^ for heart failure with ejection fraction 40% |
|  |  |

^a^At-risk drinking was defined as >14 drinks per week for men <65 years or >7 drinks per week for others. Binge drinking was defined as >4 drinks per occasion for men <65 years or >3 drinks for others.

^b^Patients where excluded from screening because of a prior diagnosis of colon cancer (n = 18) or breast cancer (n = 17)

^c^Indications for influenza immunization for <65 years: living in a nursing home, chronic cardiovascular disease, chronic obstructive pulmonary disease, renal failure, diabetes, immunosuppression, hemoglobinopathy

^d^These indicators with lower inter-rater reliability (kappa<0.6) were excludedin a sensitivity analysis

e Angiotensin-receptor blocker was added according to Joint National Committee 7 guidelines

^f^Microalbuminuria was added according to American Diabetes Association guidelines

gWhen care was contraindicated, the patient was not counted as eligible, thus reducing the denominator
