# Supplementary material for: Multimorbidity and Quality of Preventive Care in Swiss University Primary Care Cohorts
Source: PLoS One. 2014 Apr 23;9(4):e96142. doi: 10.1371/journal.pone.0096142 (PMC3997570; doi:10.1371/journal.pone.0096142)
Supplement: Table S2 — Weighted index of the Charlson index. (DOCX) [file pone.0096142.s002.docx]

**Table S2**. Weighted index of the Charlson index (based on original publication^a^)

| **Assigned weights for disease** | **Conditions** |
| --- | --- |
| 1 | Myocardial infarct |
|  | Congestive heart failure |
|  | Peripheral vascular disease |
|  | Cerebrovascular disease |
|  | Dementia |
|  | Chronic pulmonary disease |
|  | Connective tissue disease |
|  | Ulcer disease |
|  | Mild liver disease |
|  | Diabetes |
| 2 | Hemiplegia |
|  | Moderate or severe renal disease |
|  | Diabetes with end organ damage |
|  | Any tumor |
|  | Leukemia |
|  | Lymphoma |
| 3 | Moderate or severe liver disease |
| 6 | Metastatic solid tumor |
|  | AIDS |
|  |  |

^a^Based on Charlson ME [20]

Assigned wights for each condition that a patient has. The total equals the score.

Example: chronic pulmonary (1) and lymphoma (2) = total score (3).
