# Supplementary material for: Multimorbidity and Quality of Preventive Care in Swiss University Primary Care Cohorts
Source: PLoS One. 2014 Apr 23;9(4):e96142. doi: 10.1371/journal.pone.0096142 (PMC3997570; doi:10.1371/journal.pone.0096142)
Supplement: Table S3 — List of 17 selected comorbidities in ambulatory medicine. (DOCX) [file pone.0096142.s003.docx]

**Table S3**. List of 17 selected comorbidities^a^ in ambulatory medicine

| **Condition** | **Prevalence, n (%)** |
| --- | --- |
| Hypertension | 743 (75.2) |
| Diabetes mellitus | 292 (29.1) |
| COPD, Asthma | 261 (26.1) |
| Depression | 197 (19.7) |
| Coronary artery disease^b^ | 190 (19.0) |
| Renal insufficience^c^ | 167 (16.7) |
| Cancer^d^ | 142 (14.2) |
| Other psychiatric diseases^e^ | 97 (9.7) |
| Stroke (or carotid endarterectomy, hemiplegia) | 86 (8.6) |
| Liver disease (cirrhosis, hepatitis B/C) | 63 (6.3) |
| Gastrointestinal disease^f^ | 52 (5.2) |
| Connective tissue disease | 51 (5.1) |
| Heart failure | 47 (4.7) |
| Peripheral vascular disease (angioplasty, foot amputation) | 37 (3.7) |
| Major neurologic disease^g^ | 29 (2.9) |
| Dementia | 24 (2.4) |
| AIDS | 13 (1.3) |
|  |  |

^a^Based on previous studies [16] and the Charlson index[20.]

^b^Coronary artery disease, angina, myocardial infarction, other coronary heart disease, coronary angioplasty, coronary bypass

^c^End-stage renal disease, dialysis, kidney transplant, diabetic nephropathy or hypertensive nephropathy

^d^Solid non-metastatic, solid metastatic cancer, leukemia, lymphoma

^e^Bipolar disorder, psychosis, schizophrenia, pervasive developmental disorder, spastic paresia

^f^Gastric ulcus or pancreatitis or Crohn's disease or ulcerative colitis

^g^Multiple sclerosis, epilepsy,medullary compression, Parkinson, Polio or spastic paresis
